# Economic Crisis Impact and Social Determinants of Perinatal Outcomes and Infant Mortality in Greece

**DOI:** 10.3390/ijerph17186606

**Published:** 2020-09-11

**Authors:** Christos Zilidis, Christos Hadjichristodoulou

**Affiliations:** 1 General Department, University of Thessaly, 41100 Larissa, Greece; 2 Faculty of Medicine, School of Health Sciences, University of Thessaly, 41100 Larissa, Greece; xhatzi@med.uth.gr

**Keywords:** perinatal outcomes, infant mortality, economic crisis, social determinants, Greece

## Abstract

(1) Background: To explore the effects of the 2008 economic crisis on maternal, perinatal and infant mortality in Greece and the socio-economic determinants associated with them; (2) Methods: The annual rates of stillbirth (SBR), perinatal mortality (PMR), infant mortality (IMR), neonatal mortality (NNMR), post-neonatal mortality (PNMR), low birth weight (LBW), and maternal mortality (MMR) were calculated for the years 2000–2016. Average Annual Percent Changes (AAPC) were calculated by the period before and after 2008. The expected rates of 2009–2016 and the observed-to-expected rate ratios (RR) were calculated. Correlation and multiple linear regression analyses were used to test the impact of socio-economic variables on health outcomes; (3) Results: A reverse in downwards trends of PNM, IMR, and NNMR is observed since 2009. All observed values of 2009–2016 were found significantly higher than the expected ones by 12–34%. All indicators except SBR were found negatively correlated with GDP and DHI. A positive correlation was found between IMR, NNMR, and LBW and long-term unemployment, and no association with public health expenditure; (4) Conclusions: Economic crisis was associated with remarkable adverse effects on perinatal outcomes and infant mortality, mainly determined by long-term unemployment and income reduction. The findings stress a need for interventions to protect maternity and child health during crises.

## 1. Introduction

Greece was severely affected by the economic crisis of 2008 and the subsequent austerity measures applied since 2011. Between 2009 and 2014, the GDP per capita reduced by 23.6% [1], while the unemployment rate from 9.5% increased to 27.5% [2]. Public health expenditure reduced by 43.8% between 2010 and 2014 [3], while the average disposable household income decreased by 24.3% [4], diminishing the availability of resources for out-of-pocket payments.

The dramatic deterioration of socio-economic conditions affected several aspects of the population’s health [5,6,7]. Adverse effects on perinatal parameters [8,9,10] and infant mortality have also been investigated [11,12,13]. Studies based on early data from Greece, had controversial findings, recording elevated stillbirth rates but not significant changes in infant mortality [8,11]. Subsequent studies observed significant adverse effects on both perinatal parameters [9,10] and infant mortality [12,13], although they did not provide an estimation of the magnitude of that impact. Data from other affected European countries also found elevated low birth weight rates in the post-crisis period [14,15], but the findings of the impact on infant mortality remained controversial [15,16,17].

Studies exploring the mechanisms through which economic conditions affect children’s health suggest that malnutrition, infections, insufficient immunization, an increase of risky behaviors of parents, as well as changes in cohort composition may explain the effects on health outcomes [18,19,20]. Other studies suggest that economic stress has direct physiological effects, including a direct impact on the levels of several hormones and biomarkers, affecting fetuses and resulting in adverse outcomes [21,22,23,24,25]. On the other hand, harsh economic conditions are associated with reduced access to appropriate healthcare and with a deterioration of the maternal, neonatal, and infant care, which may be responsible for the deterioration of the corresponding health outcome [18,26,27,28].

Focusing on Greece, several studies found increased levels of anxiety and stress in the Greek population after the economic crisis [29,30,31], as well as significant changes in health behaviors and self-rated health and mental health [12,31,32]. Although none of the studies was specifically oriented to investigate stress in pregnant women or the psychological and health conditions in families with babies, these studies support the likelihood that economic stress may have affected the health of pregnant women and infants.

The socio-economic determinants of the observed effects have little been studied. In times of economic growth, socio-economic factors seem to be in developed countries of less importance for perinatal and infant health [33,34,35], but it is questionable whether that is true in times of financial crises. Studies from the USA and Argentina documented an increased risk of adverse birth outcomes in deprived and unemployed populations during the recession [36,37]. In Europe, a study on the effects of austerity policies applied in several countries found a positive association of these policies with elevated low birth weight rates in Greece, Portugal, and Spain [15]. Another study exploring the association of perinatal outcomes with the Human Development Index in Spain found that poor education and material deprivation worsened the negative impact of recession [38]. These studies provide indirect evidence about the impact of financial crises on perinatal outcomes and infant health. However, the direct association of the particular health outcomes with the main socio-economic effects of a financial crisis, including income reduction and unemployment, have not been extensively investigated.

This study aims to explore the effects of the 2008 economic crisis on perinatal outcomes, maternal and infant mortality in Greece and the possible correlation of these effects with critical socio-economic variables affected by the crisis.

## 2. Materials and Methods

*Study design.* This is an ecological study, using longitudinal data and applying a quasi-experimental design. We calculated and compared the health outcomes of the “exposed” to the 2008 financial crisis period with the ones of the previous “non-exposed” to the crisis period. The method uses as a control group in the same population before the impact of the financial crisis.

*Source of data*. The study is based on data obtained from the national vital statistics of Greece, including all live births, stillbirths, birth weight of live births, perinatal deaths, infant deaths by age, and maternal deaths, from 2000 to 2016. For international comparisons, we used data of the years 1996–2016, obtained from the World Health Organization (WHO) European Health for All Database [39].

*Indicators*. Considering that economic crisis may affected at a different degree the various parameters of perinatal and infant health, we calculated the annual rates of seven indicators: (i) Stillbirth rate (SBR) defined as the number of stillbirths per 1000 births; (ii) perinatal mortality rate (PMR), defined as the number of stillbirths and infant deaths within 7 days from birth per 1000 births; (iii) infant mortality rate (IMR), defined as the number of deaths within 365 days from births per 1000 live births; (iv) neonatal mortality rate (NNMR), expressing as the number of deaths within 28 days from birth per 1000 live births; (v) post-neonatal mortality rate (PNMR), defined as the number of deaths within 28 to 385 days from birth per 1000 live births; (vi) low birth weight rate (LBW), defined as the number of births with a birth weight less than 2500 gr, regardless of gestational age, per 100 live births; (vii) maternal mortality rate (MMR), representing the number of maternal deaths within 42 days of pregnancy termination due to complications of pregnancy, childbirth, and the puerperium per 100,000 live births.

*Period identification*. We applied Jointpoint regression analyses to identify the point of manifestation of the crisis effects on each of the study indicators. The Jointpoint regression identified 2008 as the turn-point of the slope in PMR, NNMR, and IMR. No turn-point was identified in the curve of SBR, PNMR, and MMR. Based on these regression results, we set the cut-point at the end of 2008 and we analyzed the data in two periods, 2000–2008 and 2009–2016

*Trend analysis*. We calculated the annual values of all indicators for the years 2000–2016 and we computed the Average Annual Percent Change (AAPC) by period, with Jointpoint Regression Program. In addition, we tested the significance of crisis impact on each indicator with Interrupted Time Series analyses (ITS), using an Autoregressive Integrated Moving Average model (ARIMA 1,0,0) with the crisis effect as a dummy variable.

*Estimation of excess mortality*. Based on the pre-crisis trends of perinatal and infant mortality indicators, we calculated the expected rates of each indicator for the post-crisis period (2009-16), using a logarithmic regression model, and we compared them with the observed ones. The model achieved an R^2^ value of 0.75 in SBR, 0.87 in PNR, 0.85 in IMR, 0.90 in NNMR, and 0.59 in PNMR. The R^2^ values in LBW and MMR were as low as 0.13 in LBW and 0.02 in MMR. Comparisons between observed and expected rates were performed by calculating the observed-to-expected rate ratios (RR) and their 95% confidence intervals (95% CI).

*International comparisons*. Using data obtained from the WHO European Health for All Database of the years 1996–2016, we explore the trends of perinatal and infant mortality in six other European countries also affected by the economic crisis; Ireland, Portugal, Spain, Italy, Hungary, and Poland. For each country, the AAPC of PMR and IMR during 2000–2008 and 2009–2016 were calculated.

*Social determinants*. We explored the correlation between the study indicators and five socio-economic variables affected by the crisis; (i) Gross Domestic Product (GDP) per capita, (ii) Disposable Household Income (DHI) per capita, (iii) unemployment rate, as a percentage of the economically active 15–64 years old, (iv) long-term unemployment rate, over twelve months of unemployment and (v) public hospital expenditure per capita.

*Statistical analysis*. Time trends and estimation of excess mortality were performed as described above. Correlation and regression analyses were based on the annual values of all dependent and independent variables, which were calculated by region, reaching 221 observations per variable. After testing for normality of variable distribution with Kolmogorov–Smirnov tests, Spearman’s correlation coefficient was used. In addition, the impact of the independent variables was tested with a multiple linear regression model. The model that better fitted was the one that included the following independent variables (i) change (%) of GDP, (ii) DHI per capita, and (iii) long-term unemployment rate. Statistical analyses were performed with the Jointpoint Regression Program and SPSS.

## 3. Results

### 3.1. Trends of Indicators

During 2000–2016, 1,779,541 live births, 7504 stillbirths, 6895 infant deaths, 29 maternal deaths, and 157,183 low birth weight births occurred in Greece. The average observed rates and trends are exhibited in Table 1, while all annual rates of all indicators are included in Supplementary Table S1.

The AAPC during the post-crisis period reversed from −5.7% to 0.9% in PMR, from −7.3% to 3.7% in IMR, and from −8.2% to 4.7% in NNMR. No significant change of the AAPC was detected in PNMR and MMR. In LBW, a step-change in 2009 was observed without further increasing trends, resulting in an average rate of 2009–2012 by 16.8% higher than 2005–2008 (95% CI 16.3–17.3%). ITS analyses found a significant change after 2008 in the slopes of PNM, IMR, NNMR, and PNMR.

### 3.2. Observed and Expected Rates

**Figure 1:**
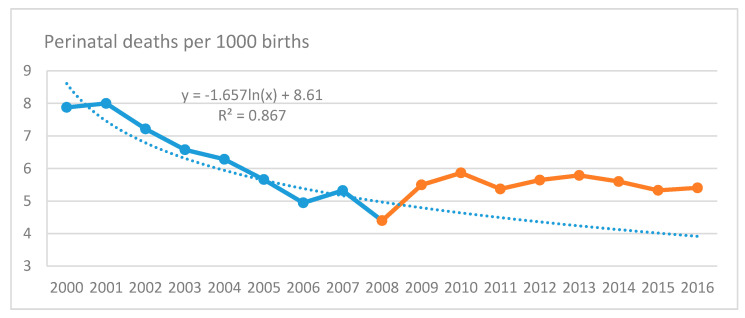

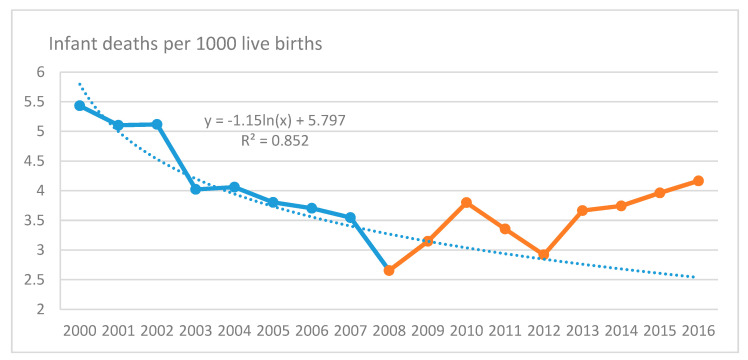
Observed and expected Perinatal Mortality and Infant Mortality rates in Greece, 2009–2016.

Based on the trends from 2000 to 2008, we calculated the expected rates of all indicators for the years 2009-16 (Figure 1). All observed values were found significantly higher than the expected ones (Table 1). The average observed rate was higher than the expected by 28% (95% CI 22–34%) in PNM, by 26% (95% CI 19–33%) in IMR, 34% (25–43%) in NNMR, 12% (2–23%) in PNMR and 12% (11–13%) in LBW. In MMR, the increase is calculated to 86%, however was found marginally not significant (95% CI −0.11% to 390%).

### 3.3. International Comparisons

Exploring the trends of perinatal mortality in other European countries affected by the financial crisis, significant changes were observed in most of them. Table 2 summarizes the AAPC by country and period, while Supplementary Table S2 provides a summary description of the main socio-economic consequences of the crisis in these countries. The negative pre-crisis AAPC of perinatal mortality reversed to positive in Portugal and Spain and reduced significantly in Ireland, Hungary, and Poland. The AAPC of infant mortality showed a significant reduction in Ireland, Portugal, Italy, and Hungary and a not significant slowdown in Spain and Poland.

### 3.4. Social Determinants

Exploring the association of the study indicators with the aforementioned socio-economic variables in Greece, a significant negative correlation of all indicators with per capita GDP and per capita DHI was found, excluding SBR (Table 3). In addition, a significant positive correlation of IMR, NNMR, and LBW with long-term unemployment rate was observed. No association was found with public hospital expenditure.

Multiple linear regression analyses found significant results in all dependent variables excluding SBR (Table 4). The B coefficient of Change (%) of GDP per capita varied from −0.375 in LBW to −0.213 in IMR, meaning that a 1% reduction of GDP per capita is associated with a rise by 0.375 of LBW, by 0.253 of PNM, and by 0.213 of IMR. The B coefficient of DHI per capita ranged from −0.258 in LBW and −0.234 in IMR to −0.122 in PNM, indicating an increase of LBW of 0.258 and IMR by 0.234 for every 1000 euros reduction of DHI per capita. The B coefficient of long-term unemployment varied from 0.332 in LBW to 0.118 in PNM, indicating the corresponding impact on each variable.

## 4. Discussion

The financial crisis of 2008 and the consequent austerity policies applied in health have been associated with a rise in mortality of several conditions [40,41]. A review of 2014 on the crisis effects on child health reported three studies with mortality outcomes from Europe, one from Canada, and one from sub-Saharan African countries [16]. The review reports that no influence was found on infant mortality in Spain and the overall child mortality in Canada [42]. However, several studies had different findings. A study based on early data from Greece reported a raised stillbirth rate but no significant changes in infant mortality [11]. Two more recent studies found an elevated risk for low birth weight [9,10] and one of them for stillbirth [9]. In Portugal, a 20–25% increase in LBW during 2008–2014 has been reported, with an average annual increase of 3.1% vs. an average decline of 4.2% from 2000–2007 [14]. A study on perinatal outcomes in European countries found a positive association of low birth weight rate with the implementation of austerity measures in Greece, Portugal, and Spain [15]. Based on the last available data, we also found a significant increase in stillbirth rate, low birth weight rate, and perinatal mortality, both in Greece and other affected European countries, which accords with the above studies. These gradually accumulated findings provide evidence that the financial crisis did affect perinatal outcomes. However, the final magnitude of the impact is not described only by the percent change of rates, but also of the duration of impact. Therefore, a meaningful question is how long that impact is extended, and the answer in that requires later data. That point is discussed below.

The findings regarding infant mortality are more controversial. According to an international report of 2014, “there is no evidence of an overall increase in infant mortality rates after the crisis in countries such as Greece, Italy, Portugal, and Spain, although the share of low birth weight babies has increased in Greece” [43]. However, some later studies provided different findings. A study on the medium-term effects of the crisis in Greece reported a rise in infant mortality [12], and another found that infant mortality was associated with the residence and Human Development Index [13]. A more recent study on infant mortality trends in European countries pointed out a change in the slope of IMR of affected countries after 2008, with a reverse in downwards trends in Ireland and Greece and a significant slowdown in Spain, Portugal, and Hungary [17]. In the present study, which is based on the last available European data, we found significant changes in the AAPC of IMR in most of the affected countries, which agree with the above findings. Assessing the overall evidence, we observe that studies based on early data found no effect or weaker effects than the later ones, probably because of a lag in effect manifestation at a statistically significant level.

Focusing on Greece, the study findings indicate an association of financial crisis with significant adverse effects on PNM, IMR, and NNMR. The correlation coefficients found do not support a very strong correlation and the results of the regression analysis show that the model can explain only 11.8% to 34.9% of the observed variability. However, although statistical analysis results impose certain reservations in supporting causality, it is remarkable that throughout the entire study period, no one indicator has returned to the last pre-crisis levels. The effects seem to be prolonged until 2016, probably because their determinants are also prolonged until that time, as discussed below. The estimation of the possible impact until 2016 shows that the observed rates in stillbirths, perinatal mortality, infant and neonatal mortality were higher than the expected ones by 25–34% over an 8-year period, and 12% in PNMR and LBW. Infant and neonatal mortality seem to be more susceptible to socioeconomic changes, although the impact on them was manifested with a delay compared with perinatal outcomes. The extension of the effects for so many years reveals a different magnitude of what a first reading of rate changes provides. In fact, the country returned at an infant health level several years back. The findings cannot be generalized; they are directly associated with the aforementioned dramatic socio-economic effects in Greece. Considering the findings in all seven countries, we should conclude that the economic crisis affected perinatal outcomes and infant health in the European countries at a different degree, according to the magnitude and duration of the socio-economic effects of the recession in each country.

Exploring the literature, two models of crisis impact on perinatal and infant health have been proposed [44]: A physiological model, which defines physiological pathways connecting economic conditions with health outcomes [21,22,23,24,25], and a healthcare model, which investigates the health outcomes in relation to the access to adequate and appropriate healthcare [26,27,28,44]. The design of the present study does not permit the investigation of possible physiological pathways connecting the economic crisis with health outcomes. It mainly permits the exploration of possible associations with socio-economic parameters that may affect health outcomes. Therefore, the main hypothesis which is investigated in the study is that socio-economic conditions produced by the prolonged economic crisis may affect perinatal and infant health, either through physiological pathways or through affecting the access to timely and appropriate healthcare. However, in the case of Greece, factors related to unhealthy behaviors seem to have a smaller impact, because unhealthy behaviors such as smoking and alcohol consumption did not increase during the crisis [12,29,30,31,32,45], while food consumption changed to a rather favorable direction [45,46]. Exploring abortions as a possible factor that may affect perinatal health, there are no data supporting an increase. Available data covering the period until 2012 show no significant changes in abortion rate [47]. Another factor that could affect outcome trends is changes in the cohort composition of pregnancies. Indeed, the age distribution of pregnant women demonstrated some significant changes. The average maternal age at birth increased gradually from 29.1 years in 2000 to 30.6 in 2008 and finally to 31.1 years in 2016 [48]. The percentage of births from mothers over 35 years, which in 2001 was 14.2%, increased to 20.9% in 2008 and finally to 30.8% in 2016 [48]. Comparing the periods 2001–8 and 2009–16, the absolute number of these births increased by 35%, while the percentage of births from mothers under 19 years reduced from 3.2% to 2.6%. Most studies, though not all, found an association between advanced maternal age and low birth weight and the risk of perinatal death [19,49,50]. However, this parameter cannot explain by itself the findings of the present study, because the increase of the average maternal age was already observed before the crisis onset when all perinatal rates were steadily declining. The further increase recorded after 2009 may have an impact, but it cannot explain the reverse of the downward trend slope.

*Social determinants*. Considering the above exploration of other possible factors that could explain the trends, the findings support an association of perinatal and infant health outcomes with the main socio-economic parameters affected by the crisis. GDP and income reduction, as well as long-term unemployment, were found to be the major socio-economic determinants of the adverse perinatal outcomes and elevated infant mortality. Although similar correlations have been found in certain studies [35,51,52], it is important to understand the nature of association in each particular case. In the case of Greece, the majority of births take place in private hospitals. According to a WHO report, even during the crisis period, and specifically during 2013–2015, a percentage of 61.6% of all births took place in private hospitals and only 38.4% in public ones [53]. Probably this is a reason for the lack of correlation between perinatal outcomes and public hospital expenditures. Nevertheless, that element makes income a critical determinant of the access to maternal and child health services for the large part of the population seeking care in the private sector. The dramatic reduction of the average household income by 24.3% [4] inevitably raised barriers in receiving the appropriate care in private settings. On the other hand, in the Greek social insurance system, health benefits are directly associated with employment status. Long-term unemployment over twelve months results in loss of health insurance benefits, including access to contracted private health services, outpatient medication, and full free access to public hospitals [54]. Consequently, the combination of high long-term unemployment and income reduction inevitably results in a restriction in health service access, for large parts of the population. Understanding that association is critical for the planning of appropriate interventions; the findings underline a need to develop programs and interventions aiming to protect maternity and child health in those parts of the population suffering high long-term unemployment or critical economic constraints. Besides, the findings question the effectiveness of the overall health insurance policy on maternity and child protection, because it reveals to be insufficient under the current social conditions. We should not overlook that even eight years after the crisis eruption, the perinatal and infant mortality rates have not yet returned to the pre-crisis levels. Considering that in 2019, unemployment remains in Greece at almost 17%, the long-term-unemployment at 13% [2], and the per capita GDP by 23% lower than in 2008 [1], we should acknowledge that the main socio-economic determinants of the adverse outcomes have not changed significantly. The perspective of a new financial crisis after the COVID-19 pandemic and the risk of a resurgence of high long-term unemployment rates restores the risk for a new worsening. Therefore, the planning of public health programs aiming to provide appropriate mother-and-child healthcare to the unemployed remains a priority. Community-based programs have been proved effective in protecting maternity and child health in vulnerable groups. A Cochrane review evaluating the effectiveness of community-based care packages to protect maternity and child, found a possible effect in terms of a reduction in maternal mortality, neonatal mortality, infant mortality, stillbirth rate, and perinatal mortality, resulting to a reduction varying from 19% to 25% [55]. The decline in perinatal and early infant mortality could be achieved by improvements in prenatal care, prevention and improvements in the medical care of infants with birth defects, access to appropriate neonatal healthcare services, and improvements in the overall infant healthcare [55,56].

On the other hand, the specific conditions in Greece set questions about the appropriateness of the model of maternal and child health protection of Greece. If health policy wants not to ignore the fact that 61.6% of births take place in private settings, it should include measures supporting pregnancy and birth independently of the ownership of health services. Limiting the support only in providing access to public hospitals, under the current conditions, seems inadequate to address the problem when 61.6% of births take place in private hospitals.

*Limitations*. The ecological nature of the study sets several limitations. Firstly, there are no data about the underlying causes of each death to directly link deaths with economic crisis effects. The findings are exclusively based on time trends of aggregated data. Secondly, the association with long-term unemployment and income reduction is also based on aggregated data and statistical correlations. We do not have individual data to attribute outcomes directly to these parameters. Thirdly, the study design does not allow identifying the mechanisms, which produce adverse effects. Fourthly, the use of projections to forecast expected values is a method that also has certain limitations. Although we selected a projection model that does not result in unacceptable values and provides sensible and conservative results, there is no way to ensure that the forecasts would happen. Besides, as in any projection, we do not know the combined impact of other not-explored determinants on the projected rates. Finally, it should be noted that the study findings are not directly comparable to those from other studies, because of different designs or methods used.

## 5. Conclusions

Considering all the above limitations, the study findings indicate a remarkable impact of the economic crisis on adverse perinatal outcomes and infant mortality, extended until 2016, and mainly determined by long-term unemployment and income reduction. The findings have obvious health policy implications, stressing a need for interventions aiming to protect maternity and child health especially in unemployed and people under critical income constrains.

## Figures and Tables

**Table 1:** Trends in perinatal and infant mortality in Greece, and expected rates 2009–2016.

	Stillbirth Rate	Perinatal Mortality Rate	Low Birthweight Rate	Maternal Mortality Rate	Infant Mortality Rate	Neonatal Mortality Rate	Post-Neonatal Mortality Rate
**Average rate**							
2001–2004	4.97	7.01	82.64	2.89	4.57	3.09	1.48
2005–2008	3.60	5.07	83.74	2.22	3.41	2.29	1.12
2009–2012	4.26	5.60	97.78	2.50	3.32	2.15	1.16
2013–2016	3.71	5.53	93.50	0.81	3.88	2.71	1.17
**AAPC (%)**							
2000–2008	−5.02 *	−5.65 *	1.87	12.46 *	−7.26 *	−8.21 *	−1.90 *
2009–2016	−1.34 *	0.91	0.35	12.46 *	3.68 *	4.66 *	−1.90 *
**ITS results**							
t	4.01	11.42	3.68	0.75	7.39	7.85	3.80
*p*	<0.01	<0.001	<0.01	-	<0.001	<0.001	<0.01
**Average observed and expected rates 2009–2016**							
Observed (O)	4.0	5.6	94.7	2.5	3.6	2.4	1.2
Expected (E)	3.2	4.3	84.7	1.3	2.8	1.8	1.0
O/E Rate Ratio	1.25	1.28	1.12	1.86	1.26	1.34	1.12
95% CI	1.19–1.31	1.22–1.34	1.11–1.13	0.89–3.90	1.19–1.33	1.25–1.43	1.02–1.23

AAPC = Average Annual Percent Change. ITS: Interrupted Time Series Analysis. * *p* < 0.05 significantly different from zero.

**Table 2:** Average Annual Percent Change (AAPC) of perinatal and infant mortality in European countries affected by the economic crisis (1996–2008, 2009–2016).

		Perinatal Mortality				Infant Mortality		
	1996–2008		2009–2015 *		1996–2008		2009–2016	
	AAPC	95%CI	AAPC	95%CI	AAPC	95%CI	AAPC	95%CI
**Ireland**	−5.64	(−7.44, −3.81)	−1.33	(−3.80, 1.19)	−4.53	(−5.40, −3.66)	−2.46	(−2.74, −2.19)
**Portugal**	−5.40	(−6.35, −4.43)	5.78	(0.13, 11.75)	−6.76	(−7.10, −6.42)	−1.42	(−1.73, −1.10)
**Spain**	−3.06	(−3.41, −2.72)	0.14	(−0.88, 1.17)	−3.01	(−3.28, −2.75)	−2.72	(−3.22, −2.21)
**Italy**	−2.90	(−4.45, −1.31)			−4.62	(−5.21, −4.03)	* −2.49	(−3.18, −1.79)
**Hungary**	−3.01	(−3.82, −2.19)	−1.23	(−3.13, 0.71)	−5.28	(−5.51, −5.06)	* −2.39	(−2.91, −1.88)
**Poland**	−6.72	(−16.56, 4.28)	−0.94	(−5.44, 3.77)	−5.63	(−6.39, −4.87)	* −4.74	(−5.65, −3.82)

* 2016 data not available.

**Table 3:** Spearman’s ρ correlation coefficient and *p*-value of the correlation between perinatal and infant mortality indicators and socioeconomic variables.

		SBR	PMR	NNMR	PNMR	IMR	LBW
Gross Domestic Product per capita							
	Spearman’s ρ	−0.122	−0.242	−0.307	−0.267	−0.372	−0.149
	*p*	0.079	0.000	0.000	0.000	0.000	0.032
Disposable Household Income per capita	Spearman’s ρ	−0.078	−0.208	−0.289	−0.194	−0.326	−0.151
	*p*	0.262	0.003	0.000	0.005	0.000	0.030
Unemployment rate	Spearman’s ρ	−0.080	0.012	0.097	−0.049	0.057	0.454
	*p*	0.252	0.859	0.164	0.483	0.416	0.000
Long-term unemployment rate	Spearman’s ρ	−0.021	0.085	0.175	0.066	0.171	0.365
	*p*	0.768	0.220	0.012	0.343	0.013	0.000
Hospital expenditure per capita	Spearman’s ρ	0.02	−0.062	−0.12	0.042	−0.097	0.049
	*p*	0.838	0.535	0.224	0.671	0.329	0.622

SBR: Stillbirth rate; PNM: Perinatal mortality rate; NNMR: Neonatal mortality rate; PNMR: Post-neonatal mortality rate; IMR: Infant mortality rate; LBW: Low birth weight rate.

**Table 4:** Multiple linear regression analyses results.

		SBR	PMR	NNMR	PNMR	IMR	LBW
Model	F-value	2.63	6.19	10.27	2.78	10.33	37.91
	*p*	0.051	<0.001	<0.001	<0.05	<0.001	<0.001
	Adjusted R^2^	0.023	0.07	0.118	0.025	0.119	0.349
Change (%) of Gross Domestic Product per capita	Standardized B coefficient	0.175	−0.253	−0.241	0.041	−0.213	−0.375
	Std. error of B	0.016	0.021	0.014	0.008	0.018	0.012
Disposable Household Income per capita	Standardized B coefficient	−0.055	−0.122	−0.182	−0.200	−0.234	−0.258
	Std. error of B	0.053	0.027	0.046	0.046	0.057	0.037
Long-term unemployment rate (%)	Standardized B coefficient	−0.200	0.118	0.203	−0.042	0.146	0.332
	Std. error of B	0.048	0.025	0.038	0.021	0.023	0.030

SBR: Stillbirth rate; PNM: Perinatal mortality rate; NNMR: Neonatal mortality rate; PNMR: Post-neonatal mortality rate; IMR: Infant mortality rate; LBW: Low birthweight rate.

## Supplementary Materials

The following are available online at https://www.mdpi.com/1660-4601/17/18/6606/s1.
