# Supplementary material for: Economic Crisis Impact and Social Determinants of Perinatal Outcomes and Infant Mortality in Greece
Source: Int J Environ Res Public Health. 2020 Sep 11;17(18):6606. doi: 10.3390/ijerph17186606 (PMC7557366; doi:10.3390/ijerph17186606)
Supplement: Supplementary file 1 [file ijerph-17-06606-s001.pdf]

Table S1. Trends in perinatal and infant mortality rates, Greece, 2000-16.

Year	Stillbirth Rate	Perinatal Mortality Rate	Infant Mortality Rate	Neonatal Mortality Rate	Post-neonatal Mortality Rate	Low Birthweight Rate	Maternal Mortality Rate
2000	5.20	7.88	5.43	3.88	1.55	80.87	0.00
2001	5.72	8.00	5.10	3.56	1.54	83.70	1.96
2002	4.90	7.22	5.12	3.50	1.62	82.53	0.97
2003	4.80	6.58	4.02	2.70	1.32	77.83	1.92
2004	4.49	6.28	4.06	2.62	1.44	86.48	1.89
2005	3.90	5.66	3.80	2.64	1.16	80.30	0.00
2006	3.34	4.95	3.70	2.51	1.20	82.76	1.79
2007	3.86	5.32	3.55	2.25	1.30	87.52	1.79
2008	3.30	4.40	2.65	1.79	0.86	84.21	0.00
2009	4.26	5.50	3.15	2.02	1.13	95.94	0.85
2010	4.34	5.86	3.80	2.47	1.32	99.88	4.36
2011	4.03	5.37	3.35	2.20	1.16	97.70	2.82
2012	4.42	5.64	2.92	1.88	1.04	97.62	1.00
2013	3.98	5.79	3.66	2.63	1.03	93.54	0.00
2014	3.82	5.60	3.74	2.57	1.17	93.69	4.34
2015	3.39	5.33	3.96	2.83	1.13	92.85	3.27
2016	3.64	5.41	4.17	2.81	1.33	93.90	3.23

Table S2. Infant mortality, perinatal mortality, average gross domestic product per capita, adjusted gross disposable income per capita, unemployment rates and public health expenditure per capita in affected European countries (2006-2016).

	Greece	Hungary	Ireland	Italy	Poland	Portugal	Spain
Real Gross Domestic product per capita (euro per capita) ([1])							
2006-8	22,427	10,400	40,100	28,480		17,110	24,193
2009-11	20,117	9,940	36,617	26,847	9,440	16,807	22,970
2012-14	17,027	10,310	37,863	25,663	10,233	16,140	22,043
2015-6	17,095	11,270	50,090	25,830	11,090	16,815	23,420
Adjusted gross disposable income of households per capita ([1])							
2006-8	18,952	11,376	20,704	21,427	10,356	16,532	18,492
2009-11	17,537	11,917	19,310	21,262	12,243	16,491	17,883
2012-14	14,842	13,110	19,116	20,780	13,990	16,433	17,527
2015-6	14,849	13,936	20,039	21,482	15,240	17,659	19,209
Unemployment rate (% of the active population) ([1])							
2006-8	8.4	7.6	5.5	6.5	10.2	8.9	9.3
2009-11	13.4	10.7	14.2	8.2	9.2	11.9	19.7
2012-14	26.2	9.6	13.7	11.8	9.8	15.4	25.1
2015-6	24.3	6.0	9.2	11.8	6.9	11.9	20.9
Long-term unemployment (% of the active population) ([1])							
2006-8	4.3	3.5	1.5	3.1	5.0	3.8	1.8
2009-11	6.1	5.0	6.4	3.9	3.0	5.4	6.8
2012-14	17.5	4.5	7.9	6.7	4.1	8.5	12.3
2015-6	17.6	2.8	4.8	6.8	2.6	6.7	10.5
Per capital public health expenditure (government schemes and compulsory contributory health care financing schemes, euro per capita) ([2])							
2006-8	…	512	…	…	…	1,040	1,384
2009-11	1,283	491	2,895	…	…	1,129	1,561
2012-14	878	499	2,894	1,834	472	985	1,426
2015-6	797	549	3,029	1,834	507	1,055	1,523

Sources: ([1]) World Bank. World Development Indicators.
https://databank.worldbank.org/source/world-development-indicators.
([2]) Eurostat. Health care expenditure by financing scheme.
https://appsso.eurostat.ec.europa.eu/nui/show.do?dataset=hlth_sha11_hf&lang=en
